# Comment on Rastogi et al. Brain Tumor Detection and Prediction in MRI Images Utilizing a Fine-Tuned Transfer Learning Model Integrated Within Deep Learning Frameworks. *Life* 2025, *15*, 327

**DOI:** 10.3390/life16040535

**Published:** 2026-03-24

**Authors:** Emmanuel Pio Pastore

**Affiliations:** Department of Biology, Ecology and Earth Science, University of Calabria, 87036 Rende, Italy; pstmnl03e19c349r@studenti.unical.it

Rastogi et al. evaluated fine-tuned transfer-learning architectures for brain tumor classification using MRI, reporting the best performance with Xception on a Kaggle-sourced dataset of tumor and non-tumor images [1]. The computational aim is clear; however, several design choices could inflate apparent performance and complicate clinical translation.

First, subject-level separation is essential to avoid inadvertent information leakage. Public MRI collections on Kaggle commonly aggregate multiple slices per subject and heterogeneous acquisitions. If random image-level splits are used, near-duplicate slices from the same person can land in both training and testing, allowing models to memorize anatomy or scanner-specific textures rather than learn generalizable pathology signatures. Reporting a patient-level split, with sequence-aware grouping when possible, or a site-withheld validation would quantify optimism arising from slice-level peeking and align the model to a plausible clinical use case [2,3].

Second, preprocessing, augmentation, and feature selection must be nested within resampling to avoid pipeline leakage. Imputation, normalization, augmentation decisions, and hyperparameter tuning (including fine-tuning schedules) should strictly be fit on training folds and then applied unchanged to held-out data. Estimating these steps on the full dataset, or tuning and evaluating within the same loop without nesting, allows outcome-related structure to bleed into validation, inflating discrimination [4]. Clear documentation of fold-wise preprocessing with fixed random seeds supports reproducibility and external appraisal [2,3].

Third, beyond accuracy on a single split, transportability under drift warrants a stricter check. MRI protocols, scanners, and patient mix evolve over time; repeated nested cross-validation plus an external or temporally separated hold-out provide a tighter stress test than one random division. Furthermore, classification probabilities should be calibrated and their uncertainty reported so that predicted risks map to observed outcomes across thresholds. Finally, decision-curve analysis connects calibrated output to action thresholds in practice (e.g., triage to advanced imaging), quantifying net benefit versus default strategies [5].

At the bedside and in multidisciplinary tumor boards, the distinction between anticipating disease and recognizing dataset-specific textures is decisive. If slice-level duplicates or acquisition-specific artifacts slip into both training and testing, performance will look high but the tool may fail on a new scanner or protocol. Building the pipeline around patient-level splits, leakage-safe nested tuning, and temporally or externally separated validation, paired with calibration and decision-analytic reporting, allows the reported accuracy to be a fair reflection of what clinicians can expect and links statistical performance to day-to-day safety.
